# Early Post-Transplant Torquetenovirus Viremia Predicts Cytomegalovirus Reactivations In Solid Organ Transplant Recipients

**DOI:** 10.1038/s41598-018-33909-7

**Published:** 2018-10-19

**Authors:** Fabrizio Maggi, Daniele Focosi, Maura Statzu, Gabriele Bianco, Cristina Costa, Lisa Macera, Pietro Giorgio Spezia, Chiara Medici, Eliseo Albert, David Navarro, Carolina Scagnolari, Mauro Pistello, Rossana Cavallo, Guido Antonelli

**Affiliations:** 1 Retrovirus Center and Virology Section, Department of Translational Research, University of Pisa, Pisa, Italy; 2 Virology Unit, Pisa University Hospital, Pisa, Italy; 3 North-Western Tuscany Blood Bank, Pisa University Hospital, Pisa, Italy; 4 Department of Molecular Medicine, Laboratory of Virology and Pasteur Institute-Cenci Bolognetti Foundation, Sapienza University of Rome, Rome, Italy; 5 Microbiology and Virology Unit, Laboratory of Virology, Azienda Ospedaliero Universitaria “Città della Salute e della Scienza” Turin, Turin, Italy; 6 Department of Microbiology, School of Medicine, University of Valencia, Valencia, Spain

## Abstract

Monitoring the human virome has been recently suggested as a promising and novel area of research for identifying new biomarkers which would help physicians in the management of transplant patients. Imbalance of the immune system in transplant recipients has a significant impact on replication of Torquetenovirus (TTV), the most representative and abundant virus of human virome. TTV kinetic was studied by real-time PCR in 280 liver or kidney transplant recipients who underwent different drug regimens to maintain immunosuppression. During one-year post-transplant follow-up, TTV viremia fluctuated irrespective of transplanted organ type but consistent with the immunosuppression regimen. TTV kinetic in patients who manifested cytomegalovirus (CMV) reactivation within the first four months post-transplant differed from that observed in patients who did not experience CMV complications. Importantly, plasma TTV load measured between day 0 and 10 post-transplant was significantly higher in CMV DNA positive than in CMV DNA negative patients. TTV viremia above 3.45 log DNA copies/ml within the first 10 days post-transplant correlates with higher propensity to CMV reactivation following transplantation. This study provides further evidence for using early post-transplant TTV viremia to predict CMV reactivation in liver or kidney transplant recipients.

## Introduction

Solid organ transplantation (SOT) is an increasingly successful treatment for patients with many acute or chronic conditions that cause irreversible and severe organ dysfunction, as it is life-saving and greatly contributes to a better quality of life in organ recipients. In the last 20 years, significant advancements in SOT have been made possible thanks to rapid expansion of immunosuppressive medications^1^. Alas, the side effects of these drugs can be severe and poorly calibrated treatments may lead to an immunosuppression status resulting either in graft dysfunction or development of microbial infections. Infections usually are the consequence of exaggerated immunosuppression and the major cause of death post-transplant^2^. To date, there is no universal biomarker to gauge immune system responsiveness in a patient treated with standard immunosuppressive regimen and assess overall integrity of the immune system. Current biomarkers (i.e. lymphocyte subset analysis, neutrophil function assay, NK activation assay, cytokine production, etc…) have poor sensitivity and specificity, do not reveal slight changes of the immune system and, above all, none of them permit evaluation of the capability of the immune system to control infections^3^. Few immunological techniques are available to measure CMV-specific T-cell immunity and identify transplant recipients at risk of complicated CMV reactivation. Such tests include enzyme-linked immunosorbent spot assay, Quantiferon-CMV, an enzyme-linked immunosorbent assays, and gamma/delta T-cell analysis. These assays require specialized personnel and equipment, are time consuming, and little suited for a routine diagnostic laboratory. Thus, there is an urgent need for a simpler, accurate, universal, and non-invasive test to precisely assess the immune system responsiveness in clinical setting, particularly in transplanted patients^4,5^.

Torquetenovirus (TTV), first identified in 1997^6^, is the prototype of a vast array of viral agents bearing similar genomes, found in humans and animals, and presently classified in *Anelloviridae* family^7^. TTV exhibits several remarkable properties, including a particularly small single-stranded circular DNA genome, a high degree of genetic heterogeneity, an uncanny ability to establish chronic infections with no clearly associated clinical manifestations, and a high worldwide prevalence regardless of age, ethnicity, sex, and socio-economic status of the population^8^. From all of these properties, it is evident that TTV has established a successful interaction with its host, even though many aspects of its life cycle and pathogenesis are still poorly understood. The interplay between TTV and host immune system is unclear too. Circumstantial evidences have recently been reported regarding the possible immunomodulatory effect of TTV and, conversely, the control of TTV replication exerted by the immune system. With regard to the latter aspect, findings have shown that TTV plasma loads tend to be higher in patients with immune system dysfunction compared to healthy controls and viremia fluctuations parallel immunity perturbations due to routine immunization, immunosuppressive medications, chemotherapy, hematopoietic stem cell transplantation, etc.^9–14^. Precise understanding of how and how much immunity modulates TTV replication is of utmost importance given the extremely high prevalence of active TTV infections in humans and the intriguing idea of using TTV viremia to gauge global immune function. In this report, plasma TTV kinetics has been studied in 280 SOT recipients during the first 12 months post-transplant to determine whether there is a relationship among TTV viremia and type of transplanted organ, immunosuppressive drug regimen, and CMV post-transplant reactivation.

## Results

### Dynamics in TTV viremia

Two hundred eighty transplant patients infected with TTV were monitored for virus levels in plasma at selected time points during the first 12 months post-transplant. Two hundred fifty-eight patients (92%) were plasma TTV positive before transplantation and, as shown in Table 1, mean TTV levels were 3.9 and 4.2 log DNA copies per ml of plasma in patients receiving kidney or liver transplant, respectively, thus confirming that TTV is particularly prevalent in the blood of patients with different clinical conditions with levels of viremia low and comparable to healthy donors in transplant candidates^13^. Plasma TTV levels remained stable or changed slightly relative to baseline up to day 20. The first significant increase (about 1 log) of mean plasma TTV load occurred at day 30 and 40 for kidney and liver transplantation, respectively. Then, TTV viremia progressively and steeply increased to a maximum of 6.9 log at day 90 and of 6.5 log at day 80 in kidney and liver transplant patients, respectively. Subsequently, TTV stabilized at levels of approximately 2–3 logs higher than those at the baseline for the remaining 9 months of observation (Table 1). TTV prevalence in 30 healthy donors was significantly lower compared to transplant patients (68% vs. 92%) and, in keeping with previous findings^14^, monthly measurement of TTV loads in plasma showed essentially stable values, with fluctuation of no more than 0.5 log copies per ml (data not shown).

**Table 1:** TTV loads in the plasma of 280 patients at selected time points post solid organ transplantation.

Days post-transplant	TTV load (log copies/ml ± SE) in patients receiving	
	Kidney transplant (n. 146)	Liver transplant (n. 134)
0	3.9 ± 0.1	4.2 ± 0.1
10	3.5 ± 0.4	4.0 ± 0.2
20	3.5 ± 0.3	4.3 ± 0.2
30	5.0 ± 0.2^a^	4.7 ± 0.1
40	5.2 ± 0.5	5.1 ± 0.3^b^
50	5.4 ± 0.4	6.0 ± 0.2
60	5.3 ± 0.4	6.1 ± 0.2
70	5.5 ± 0.6	6.0 ± 0.4
80	6.5 ± 0.6	6.5 ± 0.3
90	6.9 ± 0.2	6.1 ± 0.2
120	5.7 ± 0.5	6.0 ± 0.2
150	6.1 ± 0.8	6.3 ± 0.4
180	6.9 ± 0.2	6.4 ± 0.2
360	5.7 ± 0.3	6.3 ± 0.3

SE, standard error.

^a,b^Statistically significant to mean TTV level at the day 0 (*p* = 0. 0058, and *p* = 0. 00012; Mann-Whitney U test).

### TTV load and immunosuppressive drugs

Table 2 summarizes median TTV levels, grouped by type of immunosuppressive treatment received. As regards the induction therapy, although anti-thymocyte globulin-based regimen was associated with higher median levels of TTV compared to basiliximab-based regimen, such difference was not statistically significant. On the contrary, maintenance immunosuppression regimen based on cyclosporine A was associated with statistically significant higher median TTV loads compared to tacrolimus (*p* = 0.016¸ Mann-Whitney test).

**Table 2:** TTV loads and immunosuppressive therapy.

Variable	No. determinations	TTV DNA (log copies/ml)		*p* value^a^
		Median	95% CI	
*Induction immunosuppression*				*0.271*
Anti-thymocyte globulin	124	5.9	5.3–6.1	
Basiliximab	676	5.3	5.3–5.6	
*Maintenance immunosuppression regimen*				*0.016*
Cyclosporine-based	186	5.7	5.3–5.9	
Tacrolimus-based	1131	5.2	5.1–5.4	

CI, Confidence Interval.

^a^Calculated by Mann-Whitney test.

Statistical analyses were also performed to establish the relationship between measured tacrolimus levels and TTV loads. A total of 2671 measurements of plasma tacrolimus levels were obtained from 207 transplant patients during the first 3 months post-transplant (on average, 13 measurements per patient). Of the total tacrolimus measurements, 2033 (76%) were found within the therapeutic range of 5–15 ng/ml, 538 (20%) below and 100 (4%) with elevated levels. The median drug level was 8.8 ng/ml during the first month, 7.4 ng/ml from months one to two, and 8.1 ng/ml between months two and three post-transplant. Data from 1131 samples, which have paired measurements of tacrolimus and TTV DNA, were used to calculate whether a correlation existed between these two parameters. Mean TTV levels did not differ when compared to tacrolimus doses within 5–15 ng/ml or beyond the therapeutic ranges (<5 and >15 ng/ml) and viral loads and drug levels were not found to correlate at any time post-transplant (data not shown).

### TTV load and CMV infection

**Figure 1:**
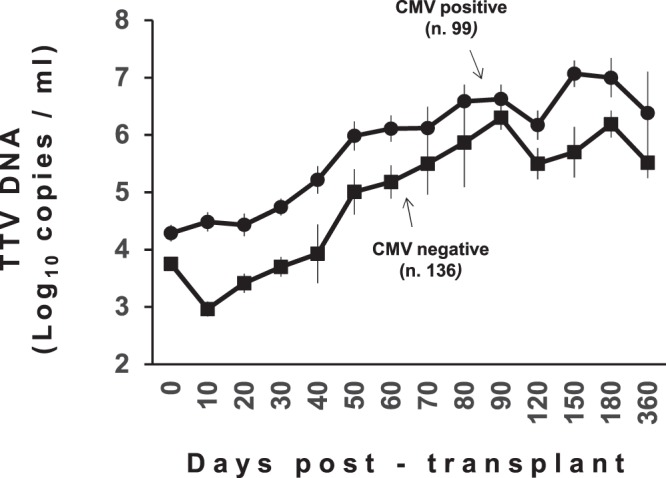
TTV DNA loads in plasma specimens of all study transplant recipients monitored for one year post-transplant and grouped according to their CMV DNA status as measured in the first four months post-transplant.

Data on monitoring of CMV infection were available for 235 (84%) subjects (145 and 90 patients receiving kidney or liver transplant, respectively) (Table 3). At admission, the mean TTV load in the 235 patients was 3.8 log copies/ml (95% confidence interval [CI], 3.7–4.0 log copies/ml). A total of 99 subjects (42%) developed CMV reactivation (35 [35%] and 64 [65%] patients receiving kidney or liver transplantation, respectively). On average, the first episode of CMV reactivation occurred within 48 days after transplantation (95% CI, 40–56 days), but in kidney transplant recipients it happened statistically later than in patients receiving liver transplant (mean 79 and 36 days, respectively; *p* < 0.001). Figure 1 summarizes the TTV kinetics in serum of kidney or liver post-transplant patients who experienced reactivation of CMV infection compared to those of patients who scored CMV real-time PCR negative throughout the follow-up. As shown, TTV loads were significantly lower during the overall observation period in CMV DNA negative patients than in CMV DNA positive patients.

**Table 3:** Demographic and clinical characteristics of the study patients.

Parameter	Patients receiving	
	Kidney transplant	Liver transplant
No. Patients	146	134
Age (mean ± SD)	62 ± 25	50 ± 27
Gender (Male/Female)	58/88	47/87
Hospital staying (days ± SD)	30 ± 21	20 ± 15
Induction immunosuppression (no. patients)		
Anti-thymocyte globulin	37	0
Basiliximab	104	121
*Total*^*a*^	141	121
Maintenance immunosuppression (no. patients)		
Cyclosporine A + MMF + steroids	5	48
Tacrolimus + MMF + steroids	139	70
*Total*	144	118
CMV DNA within four months post-transplant (no. patients)		
Positive	35	64
Negative	110	26
*Total*	145	90

MMF, *mycophenolate mofetil*.

^a^Total number of patients for which the parameter was available.

**Figure 2:**
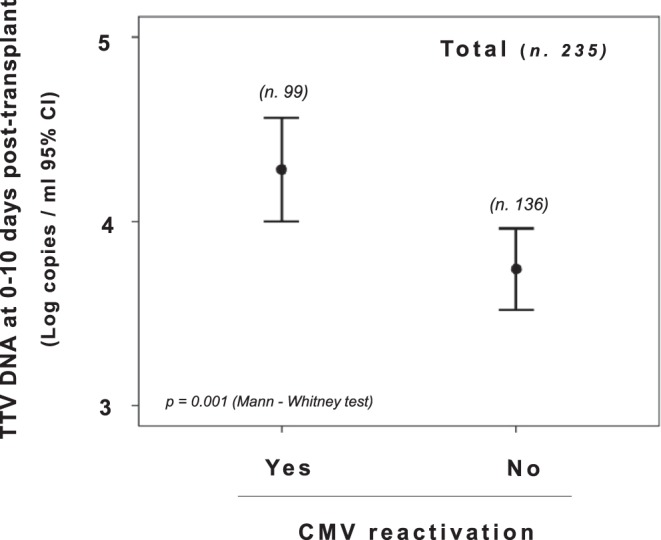
Comparison of TTV levels in all study transplant recipients monitored between 0 and 10 days post-transplant and grouped according to their CMV DNA status as measured in the first four months post-transplant.

**Figure 3:**
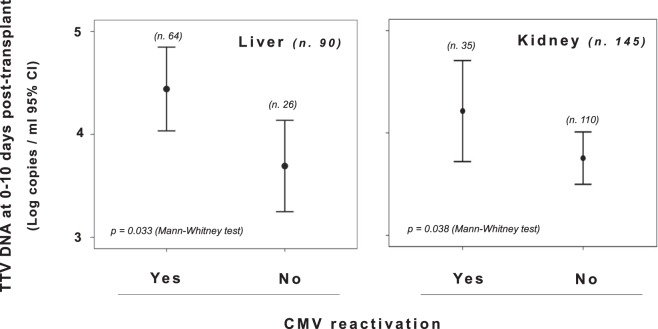
Comparison of TTV levels in liver and kidney transplant recipients monitored between 0 and 10 days post-transplant and grouped according to their CMV DNA status as measured in the first four months post-transplant.

Again, when measured between 0 and 10 days after transplantation, TTV levels were significantly higher in the CMV DNA positive patients (mean, 4.2 log copies/ml; 95% CI, 4.0–4.5 log copies/ml) than in the CMV DNA negative patients (mean, 3.7 log copies/ml; 95% CI, 3.5–3.9 log copies/ml) (Fig. 2). This finding was also confirmed by excluding from the analysis patients who were treated with anti-viral prophylaxis according to the risk of CMV infection as established from serology status of donor and recipients (data not shown). A different value of early TTV loads was also found in patients treated with CMV prophylaxis alone (mean, 4.8 and 3.8 log TTV DNA copies/ml in CMV positive and negative patients, respectively). Similar differences were observed when subjects were stratified by type of transplanted organ (Fig. 3). Of interest, when the variables potentially able to influence CMV reactivation in transplant patients (i.e. use of anti-CMV prophylaxis, CMV negative serostatus, mean tacrolimus levels at one-month post-transplant, and TTV load at 0–10 days post-transplant) were examined by regression analysis, TTV load was the only variable found to be associated with CMV reactivation (*p* < 0.05, Table 4).

**Table 4:** Odds ratios (ORs) for having CMV reactivation within four months post-transplant for 4 independent variables.

Variable	OR	OR (95% CI)	*p* value
TTV loads at day 0–10 post-tranplant	1.5	1.0–2.3	0.039
Use of anti-CMV prophylaxis	0.4	0.1–1.2	NS
CMV negative serostatus	0.8	0.2–3.1	NS
Mean Tacrolimus levels at month 1 post-transplant	1.0	0.8–1.3	NS

CI, Confidence Interval.

NS, not significant.

**Figure 4:**
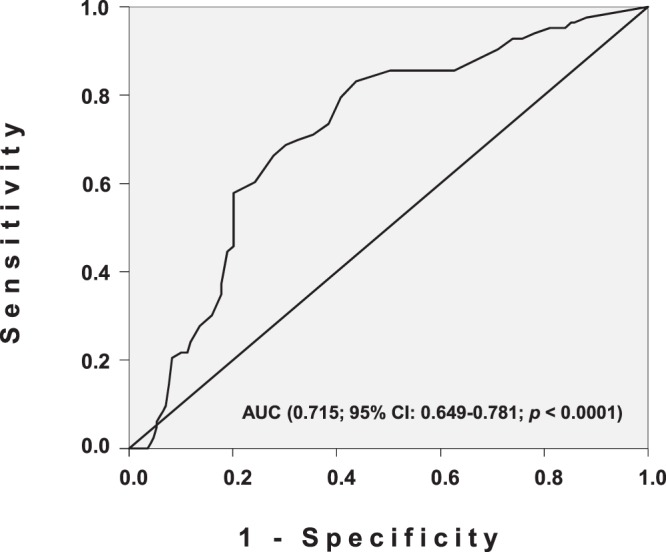
Receiver operating characteristic (ROC) curve for distinguishing the optimal cutoff value for TTV load.

The TTV threshold above which the probability to experience a CMV reactivation is difficult to establish with certainty. However, following a receiver operating cheracteristic (ROC) analysis and calculation of area under the curve (AUC) (0.715; 95% CI: 0.649–0.781; *p* < 0.0001), the optimal cutoff value for TTV load was determined at 3.45 log DNA copies/ml (sensitivity: 83.1%, specificity: 56,2%) (Fig. 4). Interestingly, no correlation was found between TTV levels measured at 0–10 days and the number of days before the diagnosis of CMV reactivation (data not shown).

## Discussion

Once thought to be present in the host only during disease, viruses have been recently demonstrated to be numerous in various compartments in healthy subjects, and the term “virome” has been coined to describe the collection of viral species present in a given human organ and as a kind of viral “flora”. This is made up of bacteriophages, endogenous retroviruses, eukaryotic viruses not associated with disease, and viruses able to cause acute, chronic or latent illness^15^. More recently, the human virome has become a promising and novel area of research for identifying new biomarkers which would help physicians in the management of diseased patients^10,16,17^. However, studying the complete spectrum of viruses forming the human virome is complicated, time-consuming, and expensive as it implies using novel molecular methods, such as next generation sequencing^18^. An important focus of virome research is the identification of single component(s) of the virome easily to monitor and serving as a diagnostic biomarker of immune system function of diseased patients.

Evidence is progressively accumulating that TTV, the most representative and abundant virus of the human virome, has an interesting interplay with cells and soluble factors known to contribute to effectiveness and/or the homeostasis of immunity^19^. It is also becoming evident that imbalance of immune status has a significant impact on TTV replication^20–24^.

The present study offers further information on this matter and provides three main findings. First, it profiles the kinetics of TTV viremia during solid organ transplantation and confirms that load changes are highly reproducible irrespective of type of transplanted organ. Since TTV replication mostly occurs in T lymphocytes^25^, immunosuppressants are thought to play a pivotal role in these changes. In line with this hypothesis, the extent of lymphocyte depletion early after induction immunosuppression (e.g. anti-thymocyte globulin vs basiliximab) has been correlated with short-term effects on viral kinetics^26^ but does not appear to affect long-term viral replication. In contrast, maintenance of immunosuppression has a prolonged effect on TTV viremia^8,26^.

Second, to the best of our knowledge, this is the largest study that attempts to correlate TTV viremia with the dose of maintenance immunosuppressant. In a series of 1131 paired samples tested for both TTV viremia and plasma tacrolimus level, we found that TTV viremia is significantly higher in patients treated with cyclosporine A compared to tacrolimus. This finding conflicts with the retrospective study carried out by Gorzer and colleagues who showed that the average burden of TTV is higher in lung transplant recipients treated with tacrolimus compared to cyclosporine A^24^. Whatever the reason for this discrepancy it should be pointed out that the Gorzer’s study used a different experimental design. The authors did not examine paired samples, but correlated TTV viremia with the mean plasma drug levels in the former month for each patient. It is worth to note that the pharmacokinetics of tacrolimus and cyclosporine are different. Tacrolimus is excreted from the body as oxidized metabolites formed during extensive metabolism by liver microsomes. This may prejudice accurate measurements of total immunosuppressive activity of the drug^27^.

In our study, we went further to ascertain possible correlations between tacrolimus plasma levels (i.e. lower, normal, and higher therapeutic ranges) and TTV viremia levels. The lack of obvious correlation may depend on the fact that the tacrolimus treatment needs prolonged periods, above three months, to impact TTV viremia. Follow-up longer than three months is warranted to shed light on this matter.

Third and most important, we corroborated the usefulness of TTV load detection to predict CMV opportunistic infections or reactivations. In the retrospective study cited above and carried out on 31 adult lung transplant recipients monitored for 2 years, Gorzer and colleagues suggested, using ROC curve analysis, that a threshold level of 9.3 log copies of TTV per ml was predictive for the development of various opportunistic infections in the following timepoint^24^. These findings were confirmed in a prospective study from the same group on lung transplant recipients^28^. This cutoff is much higher than the 3.45 log cutoff we found in our study. This difference can be due to different reasons: first, the degree of immunosuppression is higher in lung compared to kidney or liver transplantation, secondly and most importantly, the cutoff in lung transplant recipients was established at steady state and for a prolonged period of time after transplantation, i.e. when TTV viremia typically reaches a very high plateau. Our study refers to earlier, typically lower, post-transplant TTV kinetics. Cutoff values at the steady state post-transplant could be profitably used for the few cases of pre-transplant TTV negative patients. In this study, only 22 out of 280 patients (8%) were TTV negative before transplantation and 20 of them became virus positive at one month post-transplant to remain TTV viremic during the overall observation period.

Although the positive and negative predictive powers are not very high, TTV viremia competes with donor/recipient CMV serostatus as the best predictor of CMV reactivation in SOT recipients, and its measure may be use as an innovative, simple and rapid biomarker, which can be alternative or additional to the few and poorly performant tests mentioned above^4,5^.

In conclusion, in the setting of SOT, TTV is quickly evolving from an orphan and insignificant viral pathogen into a marker of immune function that has the potential to predict opportunistic infections^10,26,29,30^ and rejection^10,28,31,32^. This marker has therefore the potential to be used as patient specific maintenance of immune suppression.

## Materials and Methods

### Study population and specimens

A total of 280 adult patients who received kidney or liver transplant between 2011 and 2016 and who had completed a follow-up of at least one year were enrolled in the study. Demographic and clinical characteristics of the study patients are summarized in Table 3. Thirty healthy blood donors served as controls. All subjects gave their written informed consent, and all research was conducted under institutional review board-approved protocols (University Hospital). All experiments were performed in accordance with relevant guidelines and regulations. Peripheral blood serum samples were obtained from patients just before transplantation and every 10 days (±5 days) within the first 3 months and then at 4, 5, 6, and 12 months (±10 days) post-transplant. Samples were stored in aliquots at −80 °C until use. Plasma levels of tacrolimus were measured for each patient across the first three months post-transplant.

### TTV DNA detection and quantification

Viral DNA was extracted from 200 µl serum samples using QIAamp DNA minikit® (Qiagen, Chatsworth, CA, USA), as specified by the manufacturer. Presence and load of TTV genome were determined in a single step TaqMan®-PCR assay as described elsewhere^14^. This assay uses primers designed on a highly conserved segment of the untranslated region of the viral genome and has therefore the capacity to detect all the species in which TTV is actually classified. The method boasts high sensitivity, as low as 10 viral genomes per ml plasma, and specificity since it does not detect the other human anelloviruses, such as torquetenominivirus and torquetenomidivirus^33,34^. The procedures used to quantitate the copy numbers and assess specificity, sensitivity, intra- and inter-assay precision, and reproducibility have been previously described^12^.

### Diagnosis of CMV reactivation

CMV reactivation was defined as the presence of viral DNA above the detection threshold within the first four months post-transplant. Viral DNA was extracted from whole blood by using the QIAsymphony SP/AS instrument (Qiagen) and CMV DNA was detected and quantitated with a commercial real-time PCR assay (CMV ELITe MGB® kit, ELITech Group, Trezzano s/n, Milan, Italy). Sensitivity of the assay is 800 viral genomes per ml of whole blood.

### Statistical analysis

SPSS software version 23 (IBM, Chicago, IL, USA) was used for statistical analysis. Fisher’s exact test was applied to evaluate the heterogeneity of contingency tables. Differences between distributions were calculated by using non-parametric Mann-Whitney U test. Correlations between continuous non-normally distributed variables were assessed using Spearman rho correlation coefficient. Regression analyses were conducted to evaluate the association between the dependent variable CMV reactivation and 4 several independent variables. The odds ratio (OR) and its 95% confidence interval (CI) were calculated. The ROC curve and the relative AUC were calculated. Youden’s index was defined for all points of the ROC curve using the maximum value of the index as criterion to select for the best TTV cut-off. All *p* values presented are based on two-tailed tests, and *p* < 0.05 was considered statistically significant.

## Data Availability

The datasets generated during and/or analysed during the current study are available from the corresponding author on reasonable request.
